# Diagnostic and Prognostic Value of Serum Golgi Protein 73 in Patients With Hepatitis B Virus‐Associated Acute‐on‐Chronic Liver Failure

**DOI:** 10.1002/iid3.70120

**Published:** 2025-02-06

**Authors:** Zheng‐ju Xu, Tao Xu, Qiao‐xia Ye, Yong‐fei Li, Tian‐huang Yang, Xiao‐man Zhang, Hui Lin, Hui‐guo Liu, Zhi‐jie Huang, Jian‐kun Shen

**Affiliations:** ^1^ The Liver Disease Center The 910th Hospital of the PLA Joint Logistics Support Force, Quanzhou Fujian China; ^2^ Department of Medical Laboratory Science Quanzhou Medical College, Quanzhou Fujian China; ^3^ Department of Pathology The 910^th^ Hospital of the PLA Joint Logistics Support Force, Quanzhou Fujian China

**Keywords:** acute‐on‐chronic liver failure, diagnosis, Golgi protein 73, hepatic necroinflammation, hepatitis B virus, prognosis

## Abstract

**Background and Aims:**

The prognosis and severity of hepatitis B virus‐associated acute‐on‐chronic liver failure (HBV‐ACLF) cannot be well‐identified by serum biomarkers. The present study aims to determine the role of serum Golgi protein 73 (GP73) in predicting the prognosis and severity of liver necrotizing inflammation induced by HBV‐ACLF.

**Methods:**

A total of 427 chronic HBV‐infected patients were included for the present study. Among these patients, 179 patients had chronic hepatitis B (CHB), 96 patients had HBV‐related liver cirrhosis (LC), and 152 patients had HBV‐ACLF. The baseline and dynamic changes in serum GP73 levels were measured and compared in CHB, LC and HBV‐ACLF patients.

**Results:**

The serum GP73 levels were significantly greater in HBV‐ACLF patients when compared to CHB and LC patients. Furthermore, serum GP73 demonstrated excellent performance in distinguishing HBV‐ACLF from CHB and LC, with an area under the curve of 0.969 and 0.824, respectively. In the logistic regression analysis, a high GP73 level was identified as an independent risk factor associated with death within 3 months, and the optimal cut‐off level was 274.59 ng/mL. The serum GP73 levels significantly decreased and remained stable at approximately 6 months for survivors.

**Conclusion:**

Serum GP73 may serve as a valuable biomarker for the diagnosis and prognosis prediction of HBV‐ACLF patients.

## Graphical abstract

This study determined the role of serum Golgi protein 73 (GP73) in the prognosis and severity prediction of liver necrotizing inflammation caused by hepatitis B virus‐associated acute‐on‐chronic liver failure (HBV‐ACLF). It was found that GP73 is associated with the short‐term and long‐term prognosis of HBV‐ACLF.

## 1 Introduction

Hepatitis B virus (HBV) infection, which persists over time, is a global public health issue that leads to serious disease consequences, including acute‐on‐chronic liver failure (ACLF). ACLF is a clinical syndrome characterized by the sudden exacerbation of chronic liver disease, presenting with symptoms, such as jaundice, worsening coagulation, and hepatic encephalopathy. ACLF progresses rapidly with poor clinical efficacy and short‐term outcomes [1, 2], making it a serious threat to life. More than 30% of patients with ACLF develop multiple organ failure, and die within 30 days after diagnosis [3]. ACLF results from liver cell necrosis, which is induced by a strong cellular immune response [4]. Therefore, it is crucial to accurately determine the severity of liver necrotizing inflammation (LNI), to assess the prognosis of ACLF. The present laboratory indicators and scoring systems correlated to LNI and ACLF prognosis include the international standardized ratio (INR), total bilirubin (TBIL), and prothrombin activity (PTA), as well as the Child‐Pugh score, model for end‐stage liver disease (MELD) score, and MELD 3.0 score [5, 6]. However, these indicators and scoring systems are not optimal for predicting the severity of LNI and ACLF prognosis. Thus, reliable biomarkers are needed to help physicians better manage ACLF.

Transmembrane glycoprotein Golgi protein 73 (GP73), with a molecular weight of 73 kDa, is primarily expressed by bile duct epithelial cells under normal conditions, with significantly lower expression in hepatocytes [7]. When liver disease occurs, serum GP73 is highly expressed [8]. Furthermore, previous studies have proposed GP73 as a novel biomarker for HBV‐related hepatocellular carcinoma (HCC) and revealed that this is markedly increased in HCC patients [9, 10]. However, recent studies have doubted the role of GP73 in the management of HCC [11], suggesting the need to explore its value in other liver diseases. The previous studies conducted by the investigators revealed the correlation between the hepatic necroinflammatory grade and serum GP73 level in chronic hepatitis B (CHB) patients [12, 13], and the liver injury induced by microwave ablation [14]. Furthermore, serum GP73 might be involved in inflammatory liver diseases [15, 16, 17]. It is known that the pathological feature of ACLF is severe LNI. To date, information on GP73 in ACLF development and progression remains limited. In the present study, the correlation between the serum GP73 level and LNI in HBV‐ACLF patients was investigated. Furthermore, the long‐term prognostic value of this marker was evaluated.

## 2 Methods

### 2.1 Subjects

A cohort of 427 consecutive patients with persistent HBV infection was included for the present retrospective study. These patients were hospitalized in the 910th Hospital of the PLA Joint Logistics Support Force between September 2012 and December 2020. Among these patients, 179 patients had CHB, 96 patients had HBV‐related liver cirrhosis (LC, 23 patients had decompensated LC), and 152 patients had HBV‐ACLF. For the 179 CHB patients, 94 cases were mild, 56 cases were moderate, and 29 cases were severe. Of the 152 patients with ACLF, 80, 51 and 21 cases were in the early, middle and late stages, respectively. All patients tested positive for HBV surface antigen (HBsAg) and hepatitis B viral DNA. Meanwhile, 45 healthy adults were enlisted as normal controls.

The diagnosis and categorization of CHB infection were based on Chinese guidelines [18]. Patients who had elevated levels of alanine aminotransferase (ALT) at the time of enrollment, and were positive for HBV DNA and HBsAg for at least 6 months were considered to have CHB. Patients who had compensation LC were diagnosed with early cirrhosis, and given a class A Child‐Pugh score. Class B or C Child‐Pugh score and severe cirrhosis are the diagnostic criteria for decompensated LC. The diagnosis and categorization of ACLF were based on Chinese guidelines [19]. The HBV‐related ACLF was categorized, as follows: type A, the prevalence of chronic non‐cirrhotic liver disease; type B, the compensatory cirrhosis typically resolved within 4 weeks; type C, decompensated cirrhosis. As previously noted, HBV‐ACLF was further tiered, as follows: early, medium, and late stage. In summary, there were no problems or any extrahepatic organ failure at the early stage (30% < PTA ≤ 40% or 1.5 ≤ INR < 1.9), while there was one extrahepatic organ failure and/or morbidity at the intermediate stage (20% < PTA ≤ 30% or 1.9 ≤ INR < 2.6). In the late stage, PTA was 20% or INR was 2.6, and there were two problems and/or failure of two or more extrahepatic organs. Exclusion criteria: (1) subjects who were pregnant; (2) subjects who received antiviral therapy before screening; (3) subjects with concomitant drug‐associated chronic liver disease; (4) subjects with HCC or obstructive jaundice, or used anticoagulants; (5) subjects with thyroid disease, diabetes‐tuberculosis, acquired immune deficiency syndrome (AIDS), autoimmune liver disease, or serious cardiac, kidney, lung, nerve, or mental disorders. Blood samples were collected from all subjects on the day of hospital admission.

The present study was approved by the Ethics Committee of the 910th Hospital of the PLA Joint Logistics Support Force (Approval no. 202379).

### 2.2 Determination of Serum GP73 Levels

The serum GP73 levels were determined using the enzyme‐linked immunosorbent assay kit (Hotgen Biotech Co. Ltd., Beijing, China), according to the product instructions. The sensitivity or lower limit of detection for the serum GP73 level was 25 ng/mL, and the linear detection range was 50–500 ng/mL. The typical cut‐off value for the general healthy population was 45 ng/mL.

### 2.3 Liver Pathology

Liver specimens were collected using 16G disposable needles (C. R. Bard Inc, Murray Hill, New Jersey, USA). Samples with a portal tract number of ≥ 6 on the section or a length of ≥ 1.5 cm were considered reliable samples for liver biopsy. The samples were stained with hematoxylin and eosin, and analyzed by reticular fiber staining. The fibrosis stage and hepatic necroinflammatory grade were assessed using the Scheuer grading system by the same board‐certified liver pathologist [20].

### 2.4 Statistical Analysis

GraphPad Prism version 5.0 (GraphPad Software Inc, La Jolla, California, USA) was used to conduct the statistical analysis. Count data was expressed in percentage (%), and continuous variables were expressed in median (centile 25, centile 75). Paired *t*‐test was used to assess the difference between paired data with a normal distribution. Analysis of variance was performed to determine the differences between various groups and subgroups when the mean of the experimental data satisfied the homogeneity of variance. Kruskal–Wallis rank sum test was performed when the mean of the experimental data did not satisfy the homogeneity of variance. Univariate analysis was performed to identify the variables that significantly differed among all groups. The diagnostic efficacy of serum GP73 was evaluated using the area under the receiver operating characteristic curve (AUC), with a 95% confidence interval (CI). Sensitivity and specificity calculations were performed to assess the comprehensive diagnostic value. A *p*‐value of < 0.05 was considered statistically significant. Ya‐ling Zhu of the Huaqiao University Department of Medical Statistics assessed the statistical methodology of the study.

## 3 Results

### 3.1 Baseline Demographics and Clinical Characteristics of the Patients

An overview of the baseline clinical and demographic data for the 427 chronic HBV patients is presented in Table 1. There was no difference in gender distribution among the four groups (*p* > 0.05). However, the difference in mean age, levels of GP73, albumin (ALB), TBIL, ALT, HBV e‐antigen (HBeAg), HBV e‐antibody (anti‐HBe), and HBV DNA levels among the four groups were statistically significant (*p* < 0.05).

**Table 1:** Demographic and clinical characteristics of all participants.

Variables	Healthy control (*n* = 45)	CHB (*n* = 179)	LC (*n* = 96)	HBV‐ACLF (*n* = 152)	*t* or *F*	*p‐*value*
Age, years	31.50 (26.00, 34.00)	32.00 (26.00,38.00)	40.00 (31.25, 47.75)	41.00 (32.00, 51.00)	26.05	< 0.001
Gender, male, (%)	43/50, 86.00%	151/179, 84.36%	85/96, 88.50%	131/152, 86.18%	0.94	0.816
GP73, ng/mL	32.20 (23.41, 49.68)	66.27 (46.79, 109.01)	142.62 (91.03, 215.38)	268.52 (214.33, 313.19)	294.69	< 0.001
ALB, g/L	43.60 (40.90, 45.55)	42.70 (40.30, 45.20)	39.55 (36.03, 43.68)	33.05 (29.20, 36.15)	226.95	< 0.001
TBIL, μmol/L	15.80 (12.70, 18.70)	17.10 (12.70, 22.20)	25.45 (16.90, 62.63)	242.770 (152.73, 360.58)	310.07	< 0.001
ALT, IU/L	25.00 (17.20, 29.35)	60.90 (36.10, 123.90)	103.55 (42.48, 359.40)	937.20 (403.30, 1736.93)	269.17	< 0.001
AST, IU/L	23.00 (17.75, 28.60	59.90 (36.10, 123.90)	102.0 (40.53, 363.98)	701.95 (260.35, 1189.20)	245.37	< 0.001
HBV DNA, log [10] IU/mL	0 (0.00)	7.14 (6.36, 7.93)	6.09 (5.03, 6.72)	6.58 (5.29, 7.70)	24.99	< 0.001
HBeAg positive, *n* (%)	0 (0.00)	117/179 (65.36%)	46/96 (47.91%)	82/152 (53.95%)	8.912	0.012
Anti‐HBe positive, *n* (%)	0 (0.00)	62/179 (32.96%)	48/96 (50.00%)	66/152 (43.42%)	6.560	0.038

Abbreviations: ALB, albumin; ALB, albumin; ALT, alanine aminotransferase; AST, aspartate transaminase; CHB, chronic hepatitis B; HBV, hepatitis B virus; HBV‐ACLF, hepatitis B virus‐associated acute‐on‐chronic liver failure; HBeAg, hepatitis B e antigen; GP73, Golgi protein 73; TBIL, total bilirubin.

* The *p‐*values refer to the comparison of results for all four groups.

### 3.2 Sensitivity and Specificity of GP73 in Diagnosing Patients With HBV‐ACLF

**Figure 1:**
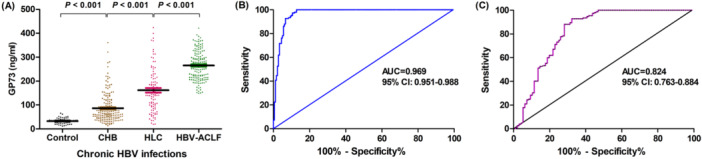
Serum GP73 levels for the different populations. (A) Comparison of serum GP73 levels in the healthy control, CHB, LC, and HBV‐ACLF groups. (B) ROC curve analysis of serum GP73 levels for discriminating patients with ACLF versus CHB. (C) ROC curve analysis of serum GP73 levels for discriminating patients with HBV‐ACLF versus LC. AUC, area under receiver operating characteristic curve; CI, confidence interval; ROC, receiver operating characteristic.

Compared to the healthy control group (32.20 [23.41, 49.68]), the mean serum GP73 levels were significantly higher in the CHB (66.27 [46.79, 109.00] ng/mL), LC (142.62 [91.03, 215.38] ng/mL), and HBV‐ACLF (268.52 [214.33, 313.19] ng/mL) groups (*F* = 294.69, *p* < 0.001; Figure 1A).

Compared to the LC and CHB groups, the serum GP73 level was significantly higher in the HBV‐ACLF group (*p* < 0.001). The results revealed that the AUC of GP73 was 0.969 (95% CI: 0.951–0.988) for discriminating patients with ACLF versus CHB (Figure 1B). At the optimal cut‐off value of 155.5 ng/mL, the sensitivity, specificity, PPV, NPV, +LR and ‐LR of serum GP73 for HBV‐ACLF diagnosis was 97.37%, 89.39%, 88.62%, 97.56%, 9.17, and 0.74, respectively. Furthermore, the AUC of GP73 was 0.824 (95% CI: 0.763–0.884) for discriminating patients with ACLF versus LC (Figure 1C). When the optimal cut‐off was set at 188.6 ng/mL, the sensitivity, specificity, PPV, NPV, +LR, and ‐LR of serum GP73 for HBV‐ACLF diagnosis was 92.76%, 67.71%, 81.50%, 85.33%, 2.86, and 0.11, respectively. Moreover, the Pearson correlation analysis revealed that there was a positive correlation between the changes in serum GP73 level and liver disease severity (healthy control to HBV‐ACLF) in chronic HBV disease progression (*r* = 0.748, *p* < 0.001).

Among the 152 HBV‐ACLF patients, 25 patients had an LC background and 127 patients had no LC background. The GP73 level for HBV‐ACLF patients with or without LC was 251.88 (213.64, 386.64) ng/mL and 263.82 (213.08, 421.47) ng/mL, respectively. There were no differences in serum GP73 levels between the two subgroups (*t* = 0.792, *p* > 0.05), indicating that the LC background did not influence the GP73 levels in HBV‐ACLF patients.

### 3.3 Serum GP73 Level Was Positively Correlated to the Hepatic Necroinflammatory Grade

Based on the liver biopsy pathology results for the 275 patients (179 CHB patients and 96 LC patients), hepatic necroinflammatory grades G1, G2, G3, and G4 were identified in 110, 73, 49, and 43 patients, respectively. The GP73 level in G1, G2, G3, and G4 patients was 54.32 (38.41, 68.56) ng/mL, 84.23 (60.02, 121.48) ng/mL, 153.14 (115.63, 195.78) ng/mL, and 199.90 (150.15, 299.13) ng/mL, respectively. There was a positive correlation between the serum GP73 level and hepatic necroinflammatory grade (*r* = 0.746, *p* < 0.001).

### 3.4 Short‐Term Prognostic Factors in Patients With HBV‐ACLF

To determine the impact of the GP73 level on the short‐term prognosis of HBV‐ACLF, the changes in serum GP73 levels were dynamically observed.

#### 3.4.1 The Relationship Between Changes in Serum GP73 and 3‐Month Survival of HBV‐ACLF Patients

A total of 152 patients were assigned into two groups, based on their survival status at 3 months after diagnosis: 115 patients were assigned to the survival group, and 37 patients were assigned to the death group.

**Figure 2:**
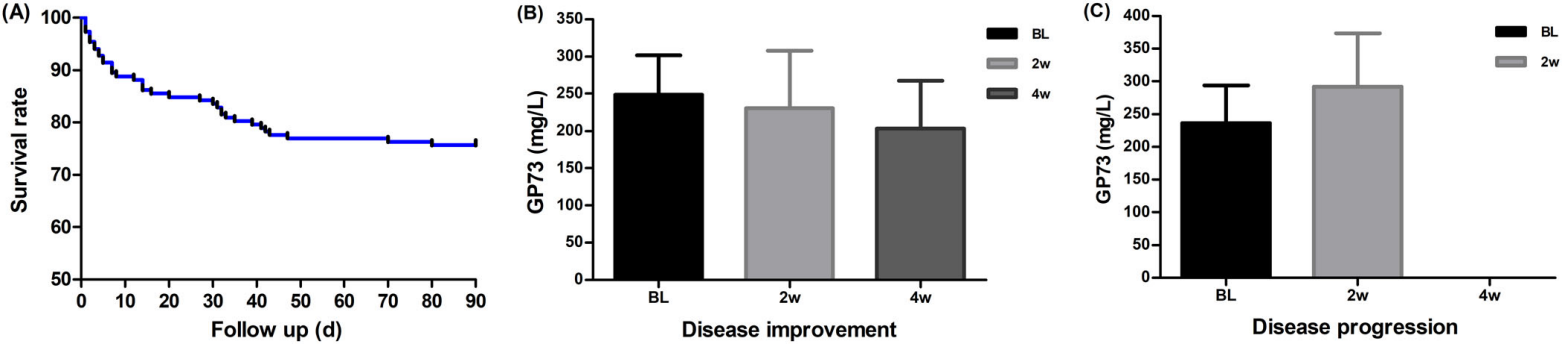
The prognosis predictive value of serum GP73 for the outcome of patients with HBV‐ACLF. (A) The 3‐month survival ratio for HBV‐ACLF. (B) Changes in serum GP73 levels for HBV‐ACLF patients with disease improvement after 4 weeks of treatment. (C) Changes in serum GP73 levels for HBV‐ACLF patients with disease progression after 2 weeks of treatment.

**Figure 3:**
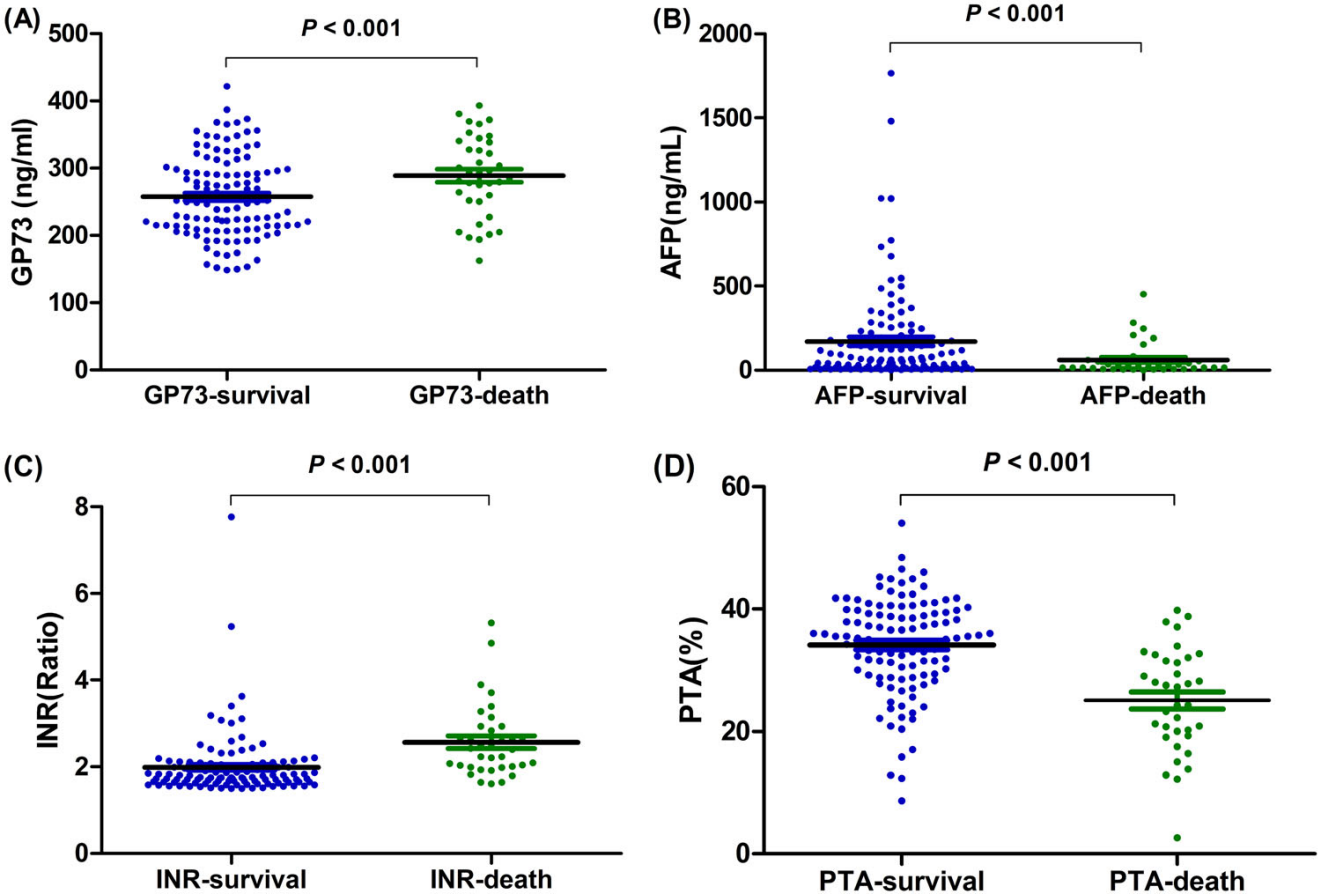
The short‐term prognostic value of serum GP73 in HBV‐associated ACLF. Comparison of serum GP73 levels (A), AFP (B), INR (C), and PTA (D) between the survival and death groups after 3 months. AFP, alpha‐fetoprotein; INR, international standardized ratio; PTA, prothrombin activity.

As shown in Figure 2A, the overall 3‐month survival rate was 75.66%. The serum GP73 levels significantly decreased in HBV‐ACLF patients who improved within 4 weeks. However, the serum GP73 levels became significantly elevated with its progression or until death within 2 weeks, as shown in Figure 2B,C. Before treatment, PTA and AFP were significantly higher in the survival group, when compared to the death group, but the serum GP73 and INR were significantly lower in the survival group. The differences were statistically significant (Figure 3A–D; all, *p* < 0.001). Furthermore, the receiver operating characteristic (ROC) curve analysis results revealed that the optimal cut‐off value for serum GP73 in predicting death due to HBV‐ACLF was 274.59 ng/mL. The AUC was 0.648, with a sensitivity and specificity of 68.75% and 60.87%, respectively.

#### 3.4.2 Logistics Regression Analysis of Factors That Affect the Short‐Term Prognosis of Patients With HBV‐ACLF

The univariate analysis results revealed significant differences in several parameters, including age and biochemical profile, as summarized in Table 2. Furthermore, TBIL, HBsAg, HBeAg, GP73, and INR significantly differed between the survival group and death group (*p* < 0.05). The multivariate analysis results identified elderly age, high GP73 level, high TBIL level, and high INR as independent risk factors associated to death within 3 months (Table 2).

**Table 2:** Logistics regression analysis of factors that affect the short‐term prognosis of patients with hepatitis B virus‐associated acute‐on‐chronic liver failure.

Variables	Survival group	Death group	Univariate analysis	Multivariate analysis		
			*p‐*value	OR	95% CI	*p*‐value
Age, years	38.00 (31.00, 44.00)	51.50 (38.50, 58.75)	< 0.001	1.083	1.025, 1.146	0.005
Gender, male, (%)	100/115 (86.97%)	28/32 (87.50%)	0.601	1.207	0.232, 6.277	0.823
GP73, ng/mL	249.19 (213.08, 298.32)	294.72 (253.66, 343.40)	0.010	1.009	1.000, 1.018	0.044
AFP, ng/mL	63.25 (13.68, 208.20)	24.73 (14.36, 68.20)	0.057	0.997	0.993, 1.000	1.002
TBIL, μmol/L	216.40 (145.40, 304.10)	378.55 (233.05, 469.18)	< 0.001	1.005	1.000, 1.010	0.044
INR	1.76 (1.64, 2.08)	2.47 (2.02, 2.95)	< 0.001	5.769	1.907, 17.454	0.002
ALT, IU/L	955.50 (406.00, 1903.20)	961.60 (372.75, 1641.43)	0.728	1.000	1.000, 1.010	0.600
AST, IU/L	688.30 (261.30, 1246.00)	818.40 (250.58, 1207.88)	0.868	1.000	0.999, 1.001	0.896
HBV DNA, log [10] IU/mL	6.39 (5.22, 7.67)	6.39 (4.59, 7.74)	0.333	1.319	0.908, 1.916	0.147
HBeAg positive, *n* (%)	45/115 (39.13%)	20/32 (62.50)	0.026	0.952	0.300, 3.024	0.933
HBsAg, log [10] IU/mL	3.72 (3.35, 3.92)	3.34 (2.64, 3.81)	0.020	0.543	0.262, 1.128	0.102

Abbreviations: AFP, alpha‐fetoprotein; ALT, alanine aminotransferase; AST, aspartate transaminase; CI, confidence interval; GP73, Golgi protein 73; INR, international standardized ratio; HBV, hepatitis B virus; HBeAg, hepatitis B e antigen; TBIL, total bilirubin; OR, odds ratio.

### 3.5 Dynamic Change in GP73 Levels During the Long‐Term Follow‐Up for HBV‐ACLF Patients

A total of 78 HBV‐ACLF patients, who underwent follow‐ups for more than 2 years, were included in the analysis. Then, the dynamic changes in serum GP73 and liver biochemical indicators (ALB, TBIL, ALT and aspartate transaminase [AST]) were determined. For survivors, the change in biochemical indicators (ALB, TBIL, ALT and AST) was monitored during the follow‐up period. After 3 months of treatment, these biochemical indicators basically returned to normal.

**Figure 4:**
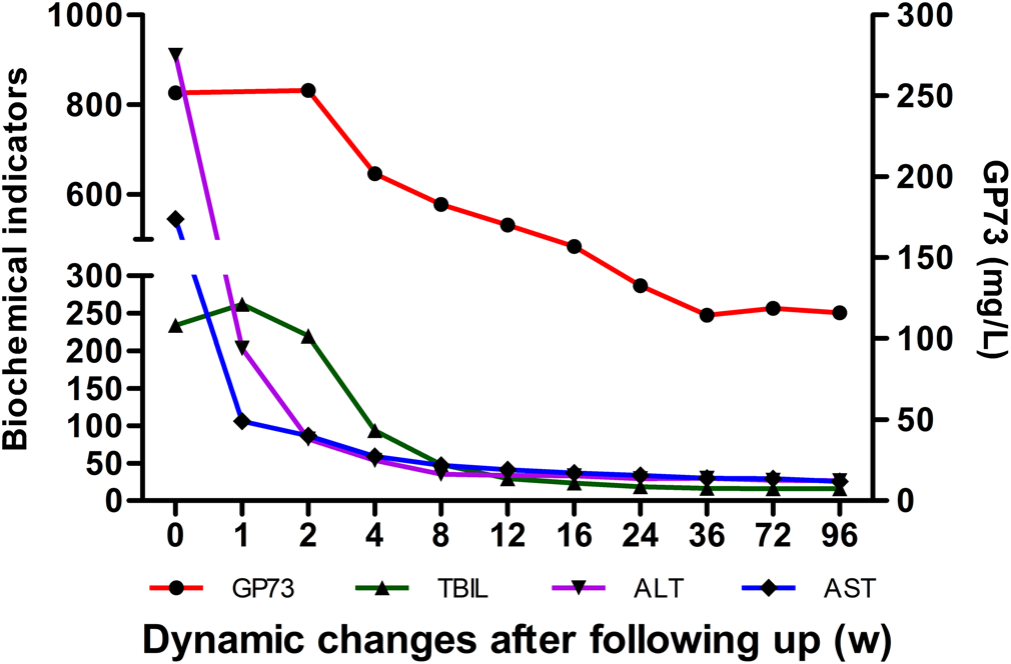
Dynamic changes in serum GP73 levels and biochemical indicators in HBV‐ACLF patients after 96 weeks of follow‐up. ALT, alanine aminotransferase; AST, aspartate transaminase; TBIL, total bilirubin; w, week.

The serum GP73 levels significantly decreased at 4, 8, 16, 24, 48, and 96 weeks after HBV‐ACLF was diagnosed. These serum GP73 levels were significantly lower, when compared to the baseline level of 251.90 ng/mL at diagnosis (Figure 4; *p* < 0.001). These levels remained stable within a narrow range of 110 ng/mL after half a year, indicating that the repair of liver tissue damage may last for approximately 6 months. In general, the dynamic changes in serum GP73 and biochemical indicators indicate that serum GP73 is a sensitive indicator that reflects LNI.

## 4 Discussion

ACLF is a unique disease characterized by organ failure, rapid onset, rapid progression, and increased short‐term mortality [4, 21]. Even liver transplantation is required for some patients with ACLF [2]. Therefore, the accurate and early evaluation of the hepatic necroinflammatory grade can help ACLF patients receive proper treatment, and improve the survival rate. Although there are various scoring models to predict the hepatic necroinflammatory grade and evaluate the prognosis of ACLF patients in clinics [22, 23], the diagnostic accuracy remains unclear. Furthermore, there is a lack of markers to precisely determine the hepatic necroinflammatory grade. Recent studies have demonstrated that the serum GP73 expressed in liver tissues is positively correlated to the hepatic necroinflammatory grade [12, 24], Thus, GP73 may be involved in the pathogenesis of inflammatory liver diseases. However, evidence for GP73 in HBV‐ACLF diagnosis and prognosis prediction remains scarce. In a recent study conducted by Atilla et al., merely a weak correlation between the serum GP73 levels, and the histological activity index scores, fibrosis stage, and AST was identified [25]. Therefore, the present study was conducted to further accumulate evidence in this field.

The present study revealed that the serum GP73 level can be used to identify the severity of LNI. Compared to CHB and LC patients, the serum GP73 levels for HBV‐ACLF patients were significantly elevated, which may be correlated to the upregulation of GP73 by inflammatory cytokines, such as interleukin‐6, after the severe immune inflammation that induces the massive and sub‐massive necrosis of liver tissues in HBV‐ACLF patients [15, 26]. This resulted in the significant release of GP73 into the circulation, which involved inflammatory damage, and the healing process of the liver. In the ROC curve analysis, the AUC for serum GP73 in distinguishing ACLF from CHB or LC was 0.969 and 0.824, respectively, demonstrating excellent performance in identifying HBV‐ACLF, and CHB or LC. Furthermore, serum GP73 exhibited promise in diagnosing HBV‐ACLF. Thus, when the serum GP73 level of CHB patients is > 155.5 ng/mL, the possibility of HBV‐ACLF should be considered. Measuring the serum GP73 level can help identify HBV‐ACLF early, allowing for the timely management of the disease. Furthermore, a high risk of death should be taken into account when the serum GP73 level is high. When the serum GP73 level was > 274.59 ng/mL, the risk of death due to HBV‐ACLF was predicted to significantly increase. Therefore, serum GP73 can be a reliable marker for evaluating the prognosis of HBV‐ACLF.

In addition to GP73, the effects of PTA and INR on the 3‐month prognostic outcome of ACLF patients were simultaneously observed. PTA and INR are important indicators that reflect the synthesis function of liver coagulation factors [27]. AFP is a particular marker used to diagnose HCC, and a crucial metric to assess the regeneration capacity of hepatocytes [28, 29]. The results revealed that the baseline PTA, INR, and AFP levels were higher in the survival group when compared to the death group, while the serum GP73 levels were lower. These results reveal that serum GP73 is a good short‐term predictor of HBV‐ACLF. That is, the higher the serum GP73 level, the worse the short‐term prognosis. The univariate analysis revealed significant differences between the survival and death groups, in terms of age, GP73, PTA, INR, AFP, and other indicators. Furthermore, the multivariate linear regression analysis results identified age, INR, GP73, TBIL, and HBsAg as independent risk factors that influenced the short‐term prognosis of HBV‐ACLF patients. Thus, a prediction model that includes all the above parameters may have better predictive ability, when compared to single indicators. Further studies are needed to verify this assumption.

In the present retrospective study, the 3‐month survival rate was 75.66%, and the short‐term survival rate was significantly higher when compared to the 50% survival rate reported in the literature [30, 31]. These present results suggest that the survival rate is significantly higher when compared to that reported in the literature. This may be correlated to the early diagnosis of HBV‐ACLF, early application of antiviral drugs, and timely control of liver failure complications in our center. Thus, early diagnosis is a key factor that affects the survival of patients [32]. To assess the effect of serum GP73 levels on short‐ and long‐term prognosis, the changes in serum GP73 levels were dynamically observed. The serum GP73 level was significantly elevated in patients who progressed or died within 2 weeks, while this significantly decreased in patients who improved within 4 weeks. After 2 years of follow‐up, the serum GP73 levels of HBV‐ACLF survivors began to significantly decline after 4 weeks. These continued to decline for half a year and remained within a narrow range of 110 ng/mL. Based on the dynamic change trend of serum GP73, the investigators speculated that although the liver function of HBV‐ACLF patients returned to normal, the repair of liver tissue damage may require a longer duration. From the study data, it was identified that serum GP73 is a good predictor for the prognosis of HBV‐ACLF.

In conclusion, serum GP73 can be used as a potential biomarker for diagnosing and predicting the prognosis of LNI induced by HBV‐ACLF. That is, the dynamic change in serum GP73 levels may be used as a prognosis indicator. The present study had some limitations. The sample size was modest for a single‐center retrospective research. Thus, a multicenter prospective investigation is required to validate these present findings. In future studies, the molecular mechanism of serum GP73 in HBV‐ACLF patients would be explored to further establish the role of this marker.

## 5 Conclusion

Serum GP73 can be used as a potential biomarker for the diagnosis and prognosis prediction of LNI induced by HBV‐ACLF. That is, the dynamic change in serum GP73 levels may be used as a prognosis indicator.

## Author Contributions

**Zheng‐ju Xu:** conceptualization, data curation, formal analysis, funding acquisition, methodology, project administration, writing–original draft. **Tao Xu:** conceptualization, data curation, investigation, project administration. **Qiao‐xia Ye:** data curation, formal analysis, investigation. **Yong‐fei Li:** data curation, formal analysis, investigation. **Tian‐huang Yang:** data curation, investigation. **Xiao‐man Zhang:** data curation, formal analysis, methodology. **Hui Lin:** data curation, methodology. **Hui‐guo Liu:** data curation,investigation. **Zhi‐jie Huang:** data curation, investigation, resources, validation. **Jian‐kun Shen:** conceptualization, formal analysis, methodology, project administration, supervision, writing–review and editing.

## Ethics Statement

The study was reviewed and approved by the Medical Ethics Committee of the 910th Hospital of the PLA Joint Logistics Support Force. All study participants or their legal guardian provided informed written consent for the personal and medical data collection prior to study enrollment.

## Conflicts of Interest

The authors declare no conflicts of interest.

## Data Availability

The data described in the study are available from the corresponding author upon reasonable request and upon completion of the required approvals. The data that support the findings of the study are available from the corresponding author upon reasonable request.
